# Estimation of the Burden of Chronic and Allergic Pulmonary Aspergillosis in India

**DOI:** 10.1371/journal.pone.0114745

**Published:** 2014-12-05

**Authors:** Ritesh Agarwal, David W. Denning, Arunaloke Chakrabarti

**Affiliations:** 1 Department of Pulmonary Medicine, Post Graduate Institute of Medical Education and Research (PGIMER), Chandigarh, India; 2 National Aspergillosis Centre, University Hospital of South Manchester, University of Manchester, Manchester Academic Health Science Centre, 2nd Floor Education & Research Centre, University of Manchester, Manchester, M23 9LT, United Kingdom; 3 Department of Medical Microbiology, Post Graduate Institute of Medical Education and Research (PGIMER), Chandigarh, India; The Australian National University, Australia

## Abstract

**Background and Objectives:**

It would be of considerable interest to clinicians if the burden of chronic pulmonary aspergillosis (CPA) and allergic bronchopulmonary aspergillosis (ABPA) in India were known. Herein, we estimate the burden of CPA following pulmonary tuberculosis (PTB), and ABPA (and severe asthma with fungal sensitization [SAFS]) complicating asthma.

**Methods:**

We used the population estimates for India from the 2011 census data. The burden of asthma was estimated using three different methods (Global Initiative against Asthma [GINA] report statement, World Health Survey [WHS] estimates, Indian study on the epidemiology of asthma and chronic bronchitis [INSEARCH]). Global and India-specific figures were used for calculating the prevalence of ABPA and SAFS. The World Health Organization estimates were used for calculating PTB rates while the frequency of CPA was assessed from a previously published scoping review. Sensitivity analysis was performed to determine the burden in various scenarios.

**Results:**

The total Indian population in 2011 was 1.2 billion. The asthma prevalence in adults was estimated at about 27.6 (range, 17–30) million. The burden of ABPA ranged from 0.12–6.09 million with different assumptions (best estimate, 1.38 [range, 0.86–1.52] million). The prevalence of SAFS was approximated at about 0.52–1.21 million (best estimate, 0.96 [range, 0.6–1.06] million). The incident TB cases were about 2.1 million while the annual incidence of CPA varied 27,000-0.17 million cases, with different estimates. If the mortality of CPA is estimated as 15% annually, the 5-year prevalence of CPA was placed at 290,147 cases with 5-year prevalence rate being 24 per 100,000.

**Conclusion:**

There is a significant burden of ABPA, SAFS and CPA in India. Prospective community-based studies are required to accurately determine the prevalence of these disorders.

## Introduction

The fungus *Aspergillus fumigatus* is a causative organism for several pulmonary diseases, the clinical presentation depending on the immune status on the host.[1] Of the various pulmonary diseases, allergic bronchopulmonary aspergillosis (ABPA) and chronic pulmonary aspergillosis (CPA) generally occur in the immunocompetent host while invasive pulmonary aspergillosis manifests in the immunocompromised host.[2] ABPA complicates the course of asthma of any severity,[3] while CPA is mostly seen arising in residual cavities of pulmonary tuberculosis (PTB).[3], [4] There is considerable morbidity associated with both ABPA and CPA. Patients with ABPA present with poorly controlled asthma (usually severe) and recurrent pulmonary infiltrates, and if untreated go on to develop extensive bronchiectasis, pulmonary hypertension and hypercapnic respiratory failure.[5] An entity related to ABPA, severe asthma with fungal sensitization (SAFS) is diagnosed by the presence of severe asthma, fungal sensitization and exclusion of ABPA.[6] Patients with CPA clinically demonstrate significant cough and dyspnea, hemoptysis (which can be life-threatening), weight loss, fatigue and progressive lung damage.[7] All these forms of chronic *Aspergillus*-related disorders respond to antifungal therapy with benefits more profound in CPA compared to allergic aspergillosis.[7]–[11].

India is a country with high burden of tuberculosis, and tuberculosis is one of the leading causes of mortality in India killing almost 1,000 individuals every day.[12] Similarly, there is a high prevalence of *Aspergillus* sensitization and ABPA complicating asthma in India.[13], [14] Thus, there is likely to be a high burden of CPA, ABPA and SAFS in India. Recently, there have been estimates of the global burden of ABPA complicating asthma and CPA as a sequel to pulmonary tuberculosis (PTB).[15], [16] It would be of great interest to clinicians and policy makers if the burden of CPA and ABPA in India is known using country-specific prevalence estimates.

In this article, we calculate the burden of ABPA (and SAFS) and CPA complicating asthma and PTB respectively, in India.

## Material and Methods

The basis for the statistical calculations has been previously published.[15], [16] We used the population estimates for India from the 2011 census data for calculating the total and the adult (≧15 years) population.[17] Briefly, the following assumptions were made.

### 

#### Burden of ABPA and SAFS

The burden of asthma in adults was calculated using three different prevalence estimates for asthma namely the Global Initiative against Asthma (GINA) report statement, the World Health Survey (WHS) estimates, and the recently published multicentric Indian study on the epidemiology of asthma and chronic bronchitis (INSEARCH study).[18]–[20] Because the GINA calculations overestimate the adult prevalence of asthma,[16] we applied the following method to estimate the adult prevalent asthma cases: Total asthma prevalence x (adult population/([0.88 × pediatric population] + adult population)). This is based on the assumption of the GINA report statement that “the mean prevalence of current wheezing in children was 88% of that recorded in adults in the countries which participated in both studies”.

The prevalence of ABPA complicating asthma globally has been estimated at 2.5% (range, 0.7–3.5%) using a scoping review methodology.[16] We not only used these figures but also performed additional modeling to accommodate several ABPA studies in India,[21]–[24] with different prevalence rates of 5%, 10% and 20%. The burden of ABPA was calculated as: adult prevalent asthma cases x prevalence of ABPA. The prevalence of severe asthma is 10% of the total asthma burden while the prevalence of fungal sensitization is about 35% (range, 30–40%).[6], [25] The prevalence of SAFS was calculated as: adult prevalent asthma cases × 0.1 × 0.35. Sensitivity analysis was performed to accommodate different prevalence figures.

#### Burden of CPA following PTB

**Figure 1:**
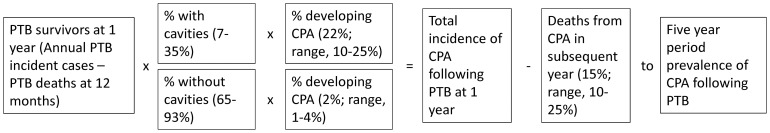
Factors used for assessing the annual incidence and 5-year period prevalence of chronic pulmonary aspergillosis (CPA) as a sequel to pulmonary tuberculosis.

The burden of CPA (Figure 1) was analyzed by using the PTB statistics and mortality rates for India published by the World Health Organization (WHO).[26] We used the WHO estimates of the number of new cases of TB (1.76 per 1000), which was then used to calculate the annual incident TB cases using the 2011 census. The prevalence of extrapulmonary TB was assumed to be 20% based on the data from the national tuberculosis control program,[12] while deaths from TB were calculated from the WHO estimates (0.22 per 1000, deaths assumed to be within 12 months of the diagnosis of TB). The extrapulmonary TB and the deaths were subtracted from the annual incident TB cases, which gave us the annual prevalent pulmonary TB cases. The frequency of pulmonary cavities after treatment for TB was estimated at 22% (sensitivity analysis was performed at prevalence of 10% and 30%).[15] The occurrence of CPA (aspergilloma) in those with pulmonary cavities was assumed to 22% (sensitivity analysis was performed at prevalence of 10% and 25%) based on previous studies.[27], [28] The prevalence of CPA in PTB without pulmonary cavities was assumed to be 2% and sensitivity analysis was performed at 1% and 4%. To calculate the burden of CPA, the 12-month mortality following CPA was assumed to be 15% (range, 10%–25%).[15] We then converted annual incident cases into five-year period prevalence using attrition rates of 10%, 15% or 25% to deduct deaths (and resection surgery) annually over the 5-year period.

All calculations were performed in Excel 2013 for Windows (Microsoft; Redmond, Washington).

An ethics clearance was not required, as the study does not involve any human subjects.

## Results

According to the 2011 census, the total Indian population was 1,210,569,573 (1.2 billion) with the adult (≧15 years) population being 838,218,964 (838 million). The prevalence of asthma was reported to be 2.05%, 3.5% and 3.3% with the INSEARCH, GINA and WHS estimates and the corresponding asthma burden in adults (≧15 years) was estimated at about 17 million (INSEARCH data), 27 million (WHS survey) and 30 million (GINA report) with various calculations (Table 1). The burden of ABPA ranged from 0.12 to 6.09 million with different inferences (Table 1). Assuming the prevalence of ABPA to be about 5% in adult asthmatics, the burden of ABPA in adult asthmatics is about 0.86 million to 1.52 million. Similarly, the prevalence of SAFS is calculated to be about 0.52–1.21 million with different assumptions. Assuming the prevalence of SAFS to be about 35%, there are about 0.6–1.06 million patients in India (Table 1).

**Table 1:** Estimated burden of allergic bronchopulmonary aspergillosis (ABPA) and severe asthma with fungal sensitization (SAFS) in adult Indian population with different rates of prevalence of ABPA in adult (≧15 years) asthmatic patients.

	INSEARCH estimates	GINA estimates	WHS estimates
Asthma prevalence	17,183,489	30,462,016	27,661,226
ABPA prevalence			
0.7%	120,284	213,234	193,629
2.5%	429,587	761,550	691,531
3.5%	601,422	1,066,171	968,143
5%	859,174	1,523,101	1,383,061
10%	1,718,349	3,046,202	2,766,123
20%	3,436,698	6,092,403	5,532,245
SAFS prevalence			
30%	515,505	913,860	829,837
35%	601,422	1,066,171	968,143
40%	687,340	1,218,481	1,106,449

The incident pulmonary TB cases in 2011 were calculated to be about 2.1 million with about 1.4 million cases surviving the illness. Assuming the prevalence of cavitation in pulmonary TB of about 22%; and, the frequency of CPA to be about 22% and 2% in tuberculous cavities and healed PTB without cavitation respectively, the annual cases of CPA was calculated at about 92,042. If the mortality of CPA is estimated as 15% annually, the 5-year prevalence of CPA was placed at 290,147 with 5-year prevalence rate being 24 per 100,000 (Table 2). The sensitivity analysis with different combinations of the occurrence of cavitation and CPA frequency is shown in Table 3. The annual incidence varied from 27,000 cases to 0.17 million cases with different presumptions (Table 3).

**Table 2:** Pulmonary tuberculosis (TB) estimates in the Indian population.

Total population in 2011	1,210,569,573
Incident TB cases	2,130, 602
Annual pulmonary TB case alive at 1 year	1,438,157
Estimated annual CPA cases after Pulmonary TB	92,042
5-year estimated CPA prevalence	290,147
5-year estimated CPA prevalence rate (per 100,000)	24

CPA: chronic pulmonary aspergillosis.

**Table 3:** Estimated burden of chronic pulmonary aspergillosis (CPA) complicating pulmonary tuberculosis (TB) in adult Indian population for different rates of annual attrition (death and/or surgical resection) and CPA frequency estimates.

CPA frequency		Annual incident CPA cases	5-year estimated CPA prevalence		
			Annual attrition of CPA		
Assuming cavitation in TB of 22%					
TB cavity	No TB cavity		10%	15%	25%
30%	4%	139,789	515,204	440,661	319,849
30%	2%	117,354	432,517	369,938	268,515
30%	1%	106,136	391,174	334,576	242,848
22%	4%	114,477	421,916	360,871	261,934
22%	2%	92,042	339,229	290,147	210,600
22%	1%	80,824	297,886	254,786	184,933
10%	4%	76,510	281,984	241,185	175,061
10%	2%	54,075	199,297	170,462	123,728
10%	1%	42,857	157,954	135,100	98,061
Assuming cavitation in TB of 10%					
30%	4%	94,918	349,830	299,215	217,181
30%	2%	69,032	254,422	217,611	157,950
30%	1%	56,088	206,718	176,809	128,334
22%	4%	44,583	164,314	140,540	102,009
22%	2%	57,526	212,018	181,342	131,625
22%	1%	83,413	307,426	262,946	190,856
10%	4%	66,155	243,821	208,543	151,369
10%	2%	40,268	148,413	126,940	92,138
10%	1%	27,325	100,709	86,138	62,522
Assuming cavitation in TB of 30%					
30%	4%	169,702	625,454	534,959	388,294
30%	2%	149,568	551,247	471,490	342,225
30%	1%	139,501	514,144	439,755	319,191
22%	4%	104,985	386,933	330,949	240,216
22%	2%	115,053	424,036	362,684	263,250
22%	1%	135,187	498,243	426,154	309,319
10%	4%	83,413	307,426	262,946	190,856
10%	2%	63,279	233,220	199,476	144,788
10%	1%	53,212	196,117	167,741	121,753

The annual incident cases of CPA were derived in pulmonary TB survivors at 1 year assuming a 22% prevalence of cavitation following pulmonary TB (sensitivity analysis at 10% and 30%). The rate of occurrence of CPA following cavitary pulmonary TB and non-cavitary pulmonary TB was assumed to be 22% (sensitivity analysis at 10% and 30%) and 2% (sensitivity analysis at 1% and 4%), respectively; which provided the annual incident CPA cases. The five year prevalence of CPA was estimated assuming an annual death rate in CPA of 15% (sensitivity analysis at 10% and 25%).

## Discussion

The results of this model, similar to many other models used for estimating the burden of other diseases, suggests a significant burden of chronic *Aspergillus*-related diseases in India. The burden of ABPA and SAFS is 0.86–1.52 million (best estimate, 1.38 million) and 0.6–1.06 million (best estimate, 0.96 million) cases respectively while the five year estimated prevalence of CPA is about 24 cases per 100,000.

We used three different methods to determine the asthma burden in India. The WHS survey is the most recent (2002–03) and employed a standardized methodology to collect data. The GINA method, on the other hand is older and used information collected from different surveys using different sampling methodologies and asthma definitions. As the GINA method overestimates the prevalence of adult asthma, we used certain assumptions to correct this, as previously described.[16] We also used asthma prevalence figures from the recently published INSEARCH study; the prevalence of asthma was lower (2.05%) with INSEARCH compared to GINA (3.5%) or WHS (3.3%) estimates as the former was a questionnaire-based study that has lower sensitivity compared to other methods used for the estimation of asthma prevalence.[19], [29] The prevalence of ABPA complicating asthma is believed to 2.5% globally however the occurrence is reported to be higher (5–20%) in India.[22]–[24] The problem with the ABPA figures from India is that the vast majority of the studies have been published from outpatient asthma clinics of tertiary care referral centers, which may not be representative of the community prevalence. The best guesstimate of the prevalence of ABPA complicating asthma is about 5%, and with this assumption the burden of ABPA is about 1 million cases. The prevalence of SAFS is also very high with almost 0.8 million cases. There is likely to be some overlap between SAFS and ABPA cases because 40% of patients with *Aspergillus* sensitization develop ABPA, and many patients with ABPA have severe asthma.[13]

The high prevalence of ABPA and SAFS has important clinical connotations. Almost one-third of the ABPA cases are initially misdiagnosed as pulmonary TB in India and thus there is a need for better recognition of this entity.[30] Also, most ABPA cases are diagnosed late in the course of the disorder manifesting with extensive bronchiectasis.[14] Hence, routine screening of all asthmatics for ABPA is recommended, especially at all special clinics and tertiary care centers. We have recently shown that *A.fumigatus* specific IgE levels are the most sensitive test in screening asthmatic patients for ABPA,[31] and this investigation should be employed in ABPA screening protocol.[32] Also, it is important to screen severe asthmatic patients for *Aspergillus* sensitization in order to diagnose SAFS, as the quality of life has been shown to improve with itraconazole therapy.[10]

The estimates of CPA prevalence were based on the incident new TB cases according to the WHO tuberculosis estimates. The data may not be robust in India as a significant proportion of TB is treated out of the national TB program. Therefore, the CPA assumptions are an underestimate of the true burden of CPA in India. In previous studies from India, *Aspergillus* antibodies were present in 26–27% of patients with TB,[33], [34] and in 23% of patients with “chronic lung diseases”, of whom 96% had prior TB at another centre.[35] The CPA prevalence is further likely to increase due to decreasing TB mortality, which would place a larger number of susceptible populations at risk. All CPA assumptions used in this analysis are based on the fact that pulmonary cavitation rates after TB (assessed on chest radiography) are about 22%.[15] Plain chest radiographs are less sensitive than computed tomography (CT) of the chest for detecting cavitation. In fact, in one study, the cavitation rates reported on computed tomography were about 35%,[36] suggesting an even higher prevalence of CPA. The occurrence of cavitation is considered central in the pathogenesis of CPA because it indicates an area of the lung with poor local host defenses and pulmonary drainage, which allows the growth of *Aspergillus*. The prevalence of CPA in non-cavitary pulmonary TB is not known, and is likely to be uncommon. Finally, we used only tuberculosis as a risk factor for CPA although several pulmonary diseases including sarcoidosis are considered as predisposing conditions for CPA.[4], [37] This is because of the overwhelming presence of TB as a predisposition for CPA, as shown recently in a study on CPA from India.[8]

In TB endemic countries including India, pulmonary symptoms and persistent or progressive pulmonary shadowing is generally attributed to relapse of PTB. Most patients, even those with sputum smear negative for acid-fast bacilli are treated for pulmonary TB. However, the possibility of CPA as a cause of pulmonary symptoms is rarely considered. It is virtually impossible to distinguish between smear-negative pulmonary TB and CPA unless one performs serological testing for *Aspergillus* IgG antibodies. Thus, it is imperative to exclude CPA in patients with suspected sputum smear-negative PTB, given the high prevalence of CPA in India.

Finally, our study is not without limitations. Our estimates are obviously crude and can be best equated to the “Fermi calculations” named after Enrico Fermi (Nobel Prize winner for physics in 1938). Calculations based on Fermi assumptions provide rough estimates, generally varying by a one-log precision. However, even rough estimates are valuable because they are the building blocks for executing future epidemiological studies.

In conclusion, a significant burden of chronic pulmonary disorders due to *Aspergillus* (ABPA, SAFS and CPA) exists in India. Early recognition and treatment with antifungal azoles has the potential to reduce the morbidity and mortality associated with these conditions. There is a need for prospective community-based and primary care clinical cohort studies to precisely define the prevalence of these disorders.
